# The immunomodulatory effects of mesenchymal stem cell-derived extracellular vesicles in Alzheimer's disease

**DOI:** 10.3389/fimmu.2023.1325530

**Published:** 2024-01-08

**Authors:** Yang Ye, Mingzhu Gao, Wentao Shi, Yan Gao, Yilu Li, Wenhui Yang, Xiaomin Zheng, Xiaojie Lu

**Affiliations:** ^1^ Research Institute for Reproductive Health and Genetic Diseases, Wuxi Maternity and Child Health Care Hospital, Wuxi School of Medicine, Jiangnan University, Wuxi, China; ^2^ Neuroscience Center, Wuxi School of Medicine, Jiangnan University, Wuxi, China; ^3^ Central Hospital of Jiangnan University, Wuxi No.2 People’s Hospital, Wuxi, China

**Keywords:** neuroinflammation, Alzheimer’s disease, mesenchymal stem cells, extracellular vesicles, central nervous immune system, peripheral immune system

## Abstract

Neuroinflammation has been identified as another significant pathogenic factor in Alzheimer’s disease following Aβ amyloid deposition and tau protein hyperphosphorylation, activated in the central nervous system by glial cells in response to injury-related and pathogen-related molecular patterns. Moderate glial cell activity can be neuroprotective; however, excessive glial cell activation advances the pathology of Alzheimer’s disease and is accompanied by structural changes in the brain interface, with peripheral immune cells entering the brain through the blood-brain barrier, creating a vicious circle. The immunomodulatory properties of mesenchymal stem cells (MSCs) are primarily conveyed through extracellular vesicles (EVs). MSC-EVs participate in chronic inflammatory and immune processes by transferring nucleic acids, proteins and lipids from the parent cell to the recipient cell, thus MSC-EVs retain their immunomodulatory capacity while avoiding the safety issues associated with living cell therapy, making them a promising focus for immunomodulatory therapy. In this review, we discuss the modulatory effects of MSC-EVs on Alzheimer’s disease-associated immune cells and the mechanisms involved in their treatment of the condition. We have found a clinical trial of MSC-EVs in Alzheimer’s disease treatment and outlined the challenges of this approach. Overall, MSC-EVs have the potential to provide a safe and effective treatment option for Alzheimer’s disease by targeting neuroinflammation.

## Introduction

Alzheimer’s disease (AD) is a neurodegenerative condition characterized by cognitive decline, memory impairments, and motor abnormalities that impact language, behavior, and visuospatial orientation (1). With the growth of the economy and the increasing average age of the population, the incidence of Alzheimer’s disease is on the rise. According to the World Health Organization’s 2019 report, there are approximately 55 million individuals worldwide affected by Alzheimer’s, a number projected to reach around 139 million by 2050 (2, 3). The exact pathogenesis of Alzheimer’s disease remains unclear, although it is widely believed to be influenced by factors such as aging, genetics, environment, and nutrition (4). Over the past few decades, the neuropathological diagnostic criteria for AD have focused on the presence of extracellular Aβ amyloid deposits known as neuritic plaques and intracellular tau protein hyperphosphorylation referred to as neurofibrillary tangles (NFTs) (5). However, therapeutic compounds tested for AD have failed to yield significant results (6), and there is mounting evidence suggesting that neuroinflammation, as a third pathological mechanism, precedes the formation of amyloid Aβ and tau protein hyperphosphorylation (7–9). Neuroinflammation refers to the presence of inflammation in the central nervous system, where glial cells are activated to respond to damage (10, 11), playing a role in neuroprotection (12). However, with the development of AD, glial cells are excessively activated, leading to an increase in pro-inflammatory cytokines, ultimately resulting in neuroinflammation and neurotoxicity (10), and further exacerbating the pathology of Aβ and tau through various mechanisms.

Mesenchymal stem cells (MSCs) are pluripotent stem cells with the capacity for self-renewal and multidirectional differentiation and are derived from numerous tissues in the body, including bone marrow, fat, muscle, lung, etc (13). Extracellular vesicles (EVs) are multifunctional intercellular messengers. They are cell-derived nano-sized double-membrane structures that contain proteins, lipids, RNA, metabolites, growth factors, and cytokines. As a cell-free bio-entity, MSC-EVs have garnered significant attention as a promising therapeutic candidate, exhibiting comparable or even superior efficacy when compared to MSCs themselves (14). In recent years, MSC-EVs have shown tremendous therapeutic potential in various diseases (15–18), including cardiovascular diseases, tumors, chronic kidney diseases, liver fibrosis, autoimmune diseases, and of course, neurological disorders such as stroke, Parkinson’s disease, and Alzheimer’s disease. In this comprehensive analysis, we delved into the alterations that occur in the innate and adaptive immune system in Alzheimer’s disease. In addition, we have explored the immunomodulatory role of MSC-EVs, especially targeting immune cells, and the relevant therapeutic mechanisms for AD. Finally, we look forward to the future with anticipation, contemplating the potentials and obstacles of MSC-EVs for clinical applications in AD.

## The immunomodulatory effects of MSC-EVs on CNS innate immune cells

It is widely believed that MSCs exert their therapeutic effects in various diseases by means of immunomodulation and tissue regeneration. This is achieved through the secretion of paracrine factors, including a class of membranous vesicles known as extracellular vesicles (EVs) (19). EVs are released into the extracellular environment by both healthy and apoptotic cells. Among the three primary subtypes of EVs, namely exosomes (exo), microvesicles (MVs), and apoptotic bodies, exosomes are the most abundant, ranging in size from 40 to 120 nm (19–21) (**Table 1**). To identify and distinguish MSC-EVs, various techniques are employed, include Electron microscopy, Nanoparticle tracking analysis (NTA), Flow cytometry, Western blotting and RNA/protein analysis (**Table 2**). MSC- EVs possess a diverse array of immunomodulatory properties, primarily targeting key components of the innate and adaptive immune systems, such as T and B lymphocytes, macrophages, dendritic cells, neutrophils, and natural killer cells (22). Many studies have confirmed that extracellular vesicles play an important role in intercellular communication. They transport bioactive lipids, mRNA, miRNA, lncRNA, and other paracrine messenger molecules, as well as genomic DNA, mitochondrial DNA, and various types of proteins (23). This process of establishing intercellular communication through the transfer of bioactive molecules can alter the activity of cells under physiological and pathological conditions (24).

**Table 1:** The characterization of different types of extracellular vesicles.

Characteristic	Exosomes	Microvesicles	Apoptotic bodies
Size(nm)	40-120	100-1000	50-4000
Morphology	Homogenous cup-shape	Heterogeneous irregular	Heterogeneous irregular
Origin	Endosomal	Plasma membrane	Apoptotic cells
Proteins	CD63, CD81, CD9, annexins, heat-shock proteins, Alix, Tsg101, clathrin, caveolins, integrins, TfRs	Integrins, flotillins, selectins, CD40, metalloproteinases	Histones
Lipids	Lysobisphosphatidic acid, cholesterol, ceramide, sphingomyelin and low concentration of phosphatidylserine	High amount of cholesterol, sphingomyelin, ceramide, high concentration of phosphatidylserine	High concentration of phosphatidylserine
Nucleic acids	mRNA and miRNA	mRNA and miRNA	mRNA, miRNA, fragments of DNA

**Table 2:** Techniques and Methods to identified MSC-EVs.

Technique	Method	Identified
Electron microscopy	allows for the visualization of the vesicles and their characteristic size and morphology	distinguish exosomes from other types of extracellular vesicles
NTA	uses laser light scattering to measure the size and concentration of particles in a sample	determine the size distribution of MSC-EVs and estimate their concentration
Flow cytometry	used to analyze the surface markers of MSC-EVs	labeling the vesicles with specific antibodies against known exosomal markers to provide information about the protein composition of the vesicles
Western blotting	used to detect specific proteins in MSC-EVs	by probing for exosomal markers to confirm the identity of the vesicles as exosomes
RNA/protein analysis	MSC-EVs can be isolated and their RNA and protein content can be analyzed	RNA sequencing and proteomics can provide information about the cargo carried by the vesicles, which can help in their identification and characterization

### Neuroinflammation and CNS innate immunity in AD

Neuroinflammation has been demonstrated to be a major factor in the pathogenesis and progression of AD, activated by damage-associated molecular patterns (DAMPs) or pathogen-associated molecular patterns (PAMPs) (7, 25). Cells contain five major pattern recognition receptors (PRRs), including Toll-like receptors (TLRs), retinoic acid-inducible gene-I (RIG-I)-like receptors (RLRs), nucleotide-binding oligomerization domain (NOD)-like receptors (NLRs), C-type lectin receptors (CLRs) and melanoma 2 (AIM2)-like receptors (ALRs), responsible for recognizing DAMPs and PAMPs, inducing inflammatory signaling pathways and immune responses that induce cell death to eliminate infected cells (26). The inflammatory response in the CNS is predominantly mediated by glial cells, including microglia and astrocytes. During the early stages of AD, microglia and astrocytes, which are innate immune cells, assume a neuroprotective role (12). However, as the disease progresses, glial cells become excessively activated and secrete substantial amounts of pro-inflammatory cytokines, thereby exacerbating neuroinflammation and further contributing to Aβ and tau protein deposition (10, 27). Consequently, this leads to synaptic damage, neuronal processes impairment, disruption of the blood-brain barrier (BBB), and infiltration of certain peripheral immune cells into the brain (28). Hence, an appropriate immune response aids in the clearance of Aβ and Tau deposits, while an excessive immune response fosters neuroinflammatory brain damage (29).

#### Microglia

As innate immune cells of the central nervous system, microglia are inactive and quiescent in the healthy brain, monitoring the surrounding neuronal environment and other glial cell communication (30). However, microglia are activated in pathological conditions such as neurodegenerative diseases, strokes and tumor invasion (31). Initially, activated microglia have an active role in the clearance of Aβ through phagocytosis; over a period of time, sustained activation also leads to a decrease in the enzymatic activity of microglia to degrade Aβ and a decrease in the efficiency of binding and phagocytosis of Aβ (32). The resultant pro-inflammatory cytokines also reduce the phagocytic activity of microglia, and they may also convert microglia to a pro-inflammatory phenotype (33, 34). In addition, pro-inflammatory microglia increase phosphorylation of tau, exacerbating the pathology of tau (35).

Microglia are able to progress towards a pro-inflammatory phenotype after sensing DAMPs and PAMPs through PRRs such as TLRs, RLRs and NLRs (36), which are at highly expressed in microglia in AD and cause inflammatory responses and pro-inflammatory cytokine secretion through PRRs signaling (37, 38). Under normal conditions, microglia clear Aβ by using surface receptors (CD14, TLR2, TLR4, α6β1 integrin, CD47) and scavenger receptors (CD36) (39, 40); with the TLR2, TLR4 and TLR4 coreceptor CD14 playing a major role (41). However, TLR2 and TLR4 in chronically activated microglia induce the production of Aβ (41) and lose the ability of Aβ elimination (42, 43). Related literature has reported that TLR2-deficient microglia cause phenotypic changes in microglia that reduce Aβ-triggered inflammatory activation and enhance phagocytosis of Aβ (44), and TLR2/4-deficient mice exhibit better neurocognitive and behavioral patterns in response to Aβ1-42 peptide than wild-type mice (45). Thus innate immune activation of microglia is implicated in AD pathogenesis.

Inflammasomes are multi-protein complexes involving intracytoplasmic pattern recognition receptors (PRRs) assembled with receptor proteins (NLR or ALR protein family), junctional proteins (ASC), and effector proteins (caspases) as the underlying structure, and are an essential component of the innate immune system, capable of recognizing PAMPs and DAMPs. A series of studies by Prof. Heneka’s team revealed that NLRP3 can be activated by persistently activated microglia in the APP/PS1 mouse model, thereby mediating caspase-1 activation and elevated expression levels of the inflammatory factor IL-1β, and that inhibition of NLRP3 activity reduces Aβ load and decreases the production of pro-inflammatory cytokines and cognitive impairment (46). Furthermore, the team revealed the pathological relationship between NLRP3 and tau and showed that inhibition of NLRP3 function was able to regulate tau kinase and phosphatase thereby reducing tau hyperphosphorylation and aggregation (47). Thus, deposition of Aβ leads to the pathological development of tau, in which NLRP3 provides a key role.

In addition, activated NLRP3 promotes the oligomerization of ASC to form large intracellular macromolecular aggregates, termed ASC spots. ASC spots have been reported to be released into the extracellular space and propagate inflammatory responses via prion-like transport mediated by phagocytosis in neighbouring macrophages (48). Friker et al. showed that in AD mice, ASC expression was increased and interacted extracellularly with Aβ to form an intensely toxic ASC-Aβ complex that was capable of causing scorch death of microglia and preventing the clearance of Aβ by microglia (49). However, the detailed molecular mechanisms underlying the release of intracellular ASC spots into the extracellular space, and their role in neuroinflammation, remain unknown.

Activation of the microglia-associated PRRs signaling pathway induce the secretion of pro-inflammatory cytokines that prompt microglia to clear Aβ, but the release of pro-inflammatory cytokine and activation of inflammasome caused by excessive microglia activation further contributes to AD pathology.

#### Astrocyte

Astrocytes are the most common glial cells in the brain (50) and play an important role in regulating blood flow, maintaining the blood-brain barrier (BBB), providing energy metabolites to neurons, regulating extracellular ion homeostasis and modulating synaptic activity (51). Astrocytes express numerous receptors for PAMPs and DAMPs known to trigger innate immune responses, particularly TLRs, including TLR4 (52), in response to activators of innate immune responses (53). In contrast, astrocytes exhibit a response in response to CNS injury and disease that is often termed astroglial cell reactivity (54). Reactive astrocytes are an integral part of the innate immunity of the central nervous system. Similar to microglia, reactive astrocytes are divided into pro-inflammatory A1 and immunomodulatory (neuroprotective) A2 subsets (55). Pro-inflammatory reactive astrocytes upregulate complement cascade genes and induce pro-inflammatory factors such as IL-1β and TNF-α, while neuroprotective reactive astrocytes upregulate and support neuronal growth with a range of neurotrophic factors (56). Professor Barres’ research has shown that reactive astrocytes A1 lose the function of resting astrocytes to form synapses and produce toxic effects on neurons. In addition, as synaptic loss is also a characteristic feature of AD, Barres et al. also found that in AD, nearly 60% of astrocytes in the prefrontal cortex (the active site of the disease) are in the A1 condition and drive the disease progression in AD due to the high toxicity of A1 to neurons and oligodendrocytes (57).

Although the innate immune sensing of astrocytes is not well understood, many studies have shown that astrocytes and microglia regulate each other’s functions by secreting cytokines. On the one hand, the inflammatory factors TNF-α, IL-1 and C1q secreted by activated microglia induce the transformation of resting astrocytes into neurotoxic reactive astrocytes A1 (57); on the other hand, the large amount of IL-3 secreted by astrocytes is able to bind to the IL-3α receptor aberrantly expressed by microglia in AD disease and is capable of regulating microglia to perform the clearance of Aβ function (58). Thus, the interaction between astrocytes and microglia may become a new therapeutic direction.

### MSC-EVs inhibit glial cell activity

As previously mentioned, the excessive activation of glial cells exacerbates the neuroinflammatory pathology of Alzheimer’s disease. Numerous *in vitro* and *in vivo* experiments have demonstrated that the extracellular vesicles derived from mesenchymal stem cells (MSCs) inhibit the activity of glial cells, thereby reducing the expression of pro-inflammatory cytokines and alleviating neuroinflammation. Mao Ding et al. discovered that extracellular vesicles from human umbilical cord MSCs regulate the levels of inflammatory cytokines by modulating the activity of microglial cells *in vitro*. Injection of extracellular vesicles derived from human umbilical cord MSCs into AD mouse models has been shown to improve cognitive impairment and promote the clearance of Aβ. Additionally, there is a decrease in the number of inflammatory microglial cells, an increase in the levels of immunoregulatory microglial cells, a reduction in the levels of pro-inflammatory cytokines (IL-1β and TNF-α) in the peripheral blood and brain of mice, and an elevation in the levels of anti-inflammatory cytokines (IL-10 and TGF-β) (59). In addition, the Mesenchymal stem cell-derived exosomes can also reduce the activity of astrocytes. In a study on exosomes derived from hypoxia-preconditioned MSCs (PC-MSCs), injection of PC-MSC exosomes significantly improved the learning and memory abilities of APP/PS1 mice compared to exosomes from normoxic MSCs. The activity of microglia and astrocytes was reduced, plaque deposition and Aβ levels were decreased, and the expression of growth-related protein 43, synaptophysin 1, and IL-10 was increased. The levels of neuroglial fibrillary acidic protein, ionized calcium-binding adapter molecule 1, TNF-α, IL-1β, as well as the activation of STAT3 and NF-κB, all sharply decreased. This may be attributed to the higher expression of miR-21 in PC-MSC exosomes (60). Some studies have indicated that the levels of miRNA-21 significantly decrease in the presence of chronic inflammation and cellular apoptosis. However, the mesenchymal stem cells in the extracellular matrix exhibit high levels of miRNA-21, which contribute to the reduction of inflammation and cellular apoptosis (61). Therefore, the extracellular vesicles released by mesenchymal stem cells containing miRNA can inhibit the activity of immune cells and induce their phenotypic transformation into anti-inflammatory. Vascular dementia (VaD) is another common cause of dementia, following Alzheimer’s disease. In the establishment of a VaD rat model through bilateral carotid artery ligation, there is an increase in inflammatory microglial cells. HUCMSC-Evs, by activating the PI3K/AKT/Nrf2 pathway, suppresses the activity of inflammatory microglial cells, inflammation, and oxidative stress, thereby protecting the neural function of VaD rats (62).

## The immunomodulatory effects of MSC-EVs on peripheral immune cells

### Peripheral immune cell infiltration in AD

As mentioned above, excessive protein deposition in AD triggers a shift in glial cells towards an inflammatory phenotype, the release of pro-inflammatory cytokines and complement, causing hyperactivation of glial cells and a vicious cycle of neurodegeneration. In this state, structural or biological changes occur at the brain interface, allowing peripheral immune cells to infiltrate the brain parenchyma through the blood-brain barrier (63), choroid plexus (64, 65) or meninges (66, 67), exacerbating the pathological development of AD. Single cell sequencing has shown that peripheral immune cells include myeloid cells such as natural killer cells (NK cells), polymorphonuclear neutrophils (PMNs), monocytes/macrophages, dendritic cells (68), and adaptive immune cells such as T cells (69) and B cells (70, 71). Due to the unclear role of dendritic cells in AD, as well as the controversial results regarding how MSC-EVs regulate B cells. Here we focused on macrophages and T cells.

#### Monocytes/macrophages

In AD, damage to the central nervous system leads to increased permeability of the BBB, favoring infiltration of peripheral monocytes. Aβ has been shown to induce the release of chemokines, such as monocyte chelator proteins (MCPs), capable of attracting monocytes. Pro-inflammatory cytokines such as IL-6 and TNF-a produced by monocytes to enhance their phagocytosis of Aβ (72). A recent study has shown that inflammatory monocytes/macrophages are elevated in cell cultures stimulated by Aβ in AD patients. These cells express TLR2, TLR4, IL-6 and CCR2, which in turn can facilitate the migration of monocytes/macrophages across the BBB into the brain. Research has shown that patients with AD and mild cognitive impairment (MCI) exhibit higher expression of TLR3 and TLR8 in monocytes/macrophages, as well as production of IL-23. Additionally, AD monocytes/macrophages also possess independent MHC-II/Aβ42 complexes. These findings suggest that monocytes/macrophages in AD exhibit inflammatory characteristics and are involved in both innate and adaptive immune responses through TLR stimulation. Furthermore, they may present Aβ peptides in an MHC-restricted manner (73). In the presence of soluble or mildly aggregated Aβ, there is an increase in T cell proliferation and pro-inflammatory cytokine secretion. These observations indicate that Aβ may not only act as an antigen but also as a more widespread positive regulator of peripheral adaptive immune responses. When activated T cells cross the blood-brain barrier and enter the brain, they can also modulate adaptive immune responses within the brain (74). In parallel, alterations in the monocyte/macrophage subpopulation were observed in AD (75, 76), but whether this alteration is due to a shift in the monocyte phenotype or the gradual death of classical monocytes remains to be further investigated.

#### T cell

Lymphocytes are an indispensable component of the adaptive immune system, and mounting evidence suggests that adaptive immune cells play a crucial role in the pathophysiology of neurodegenerative diseases such as Alzheimer’s disease (AD). T cells infiltrate the central nervous system during the onset of AD, promoting neuroinflammation (69, 77–79). On the one hand, helper T cells cross the blood-brain barrier and interact with glial cells, triggering immune and inflammatory responses, ultimately leading to neuroinflammation and neuronal damage. Browne et al. (80). found a significant presence of T cells, particularly Aβ-specific Th1 cells, in the brains of APP/PS1 mice, which increased activation of microglia and Aβ deposition through the production of IFN-γ, resulting in cognitive impairment. *In vitro* experiments conducted by McQuillan et al. (81). also demonstrated that Aβ-specific Th1 and Th17 cells induce glial cells to produce pro-inflammatory cytokines, while Th2 cells attenuate this effect. The aforementioned study elucidates that the regulation of T cell activation on microglia is contingent upon their cellular phenotype. Furthermore, T cell activation can also promote the activation and proliferation of glial cells, thereby exacerbating the inflammatory response. Earlier work by Yong et al. (82) demonstrated that IFN-γ produced by T cells induces proliferation of astrocytes *in vitro* and facilitates reactive astrogliosis in the brain. Currently, IL-17 produced by Th17 cells has been repeatedly confirmed as an effective stimulant for astrocytes. IL-17 stimulation activates inducible nitric oxide synthase (83), regulates the expression of macrophage inflammatory protein-1α (MIP-1α) through the PI3K/Akt and NF-κB pathways (84), and enhances the IL-6 signaling pathway in astrocytes (85). On the other hand, the infiltration of cytotoxic T cells is associated with the deterioration of AD (69, 77, 78). A recent study discovered the presence of clonally expanded CD8+ cells in the cerebrospinal fluid of AD patients, with TEMRA being the predominant subset (69). These cells are associated with immune memory and can release inflammatory factors and cytotoxic molecules. Furthermore, the cytotoxic effector genes of these cells are highly expressed in the hippocampus of AD patients. Additionally, the levels of TEMRA cells in the peripheral blood of AD patients show a negative correlation with cognitive levels, as well as a negative correlation with central memory T cells (TCM) and effector memory T cells (TEM). This suggests that adaptive immune cells may also play a role in Alzheimer’s disease, and CD8+ T cells may impact neurodegeneration and/or cognitive impairment in AD.

#### Other immune cells

NK cells are potent cytotoxic effectors against infected pathogens and tumor cells (86, 87). They play a crucial role in bridging the innate and adaptive immune systems by secreting cytokines and interacting with other immune cells. Compared to healthy elderly individuals of matching age, the distribution of NK cells in AD patients remains unchanged. However, in the early stages of AD, specifically in cases of amnestic mild cognitive impairment (aMCI), NK cells are activated and exhibit stronger activity (88). For instance, increased production of granule enzyme B and pro-inflammatory cytokines (TNFα, IFNγ) has been observed in aMCI subjects, contrasting with NK cells in confirmed cases of mild AD (mAD) (87). The activated state of NK cells may be a congenital immune response to cope with unidentified challenges, which may include viral or microbial agents. Furthermore, this activation state may contribute to the occurrence of neuroinflammation. Therefore, the protective role of NK cells may no longer be effective in the progression from aMCI to AD, and NK cells could potentially be considered as biomarkers for the early stages of AD.

At the same time, polymorphonuclear neutrophils (PMN), as frontline immune cells, also participate in the early stages of AD. The functional changes of these cells during different stages of AD pathology may be associated with pathological stimuli (89). CD177 expression was increased in mAD but not in healthy individuals or aMCI patients. Expression of CD14 and CD16 was lower in the PMN of patients with mAD compared with controls, whereas it was unchanged in patients with aMCI. Only the PMN of aMCI patients expressed lower levels of CD88. The production of inflammatory cytokines (TNFα, IL-6, IL-1β, IL-12p70) and chemokines (MIP-1α, MIP-1β, IL-8) in response to LPS stimulation was very low in patients with aMCI and virtually absent in patients with mAD. TLR2 is only expressed at lower levels in aMCI. We therefore suggest that since AD may be the result of a pathogen challenge, neutrophils at the front line will fight the pathogen and instruct other immune cells to intervene. In this way, neutrophils may be involved in the earliest stages of AD pathogenesis.

Although the extracellular vesicles of MSCs have shown potential therapeutic effects in immune regulation, further research is needed to understand their role in modulating immune cells in AD. AD is a neurodegenerative disorder that is associated with abnormal activation of the immune system and inflammatory responses. Therefore, understanding the regulatory effects of MSCs extracellular vesicles on immune cells in Alzheimer’s disease is of great significance in uncovering the mechanisms of disease progression and developing new treatment strategies.

## The mechanism of MSC-EVs in treating AD

The immunomodulatory effects of MSC-EVs on immune cells mainly manifest in inhibiting glial cell activity, reducing the expression of inflammatory factors, thereby alleviating neuroinflammatory reactions; inhibiting the proliferation and differentiation of lymphocytes, promoting the differentiation of lymphocytes into anti-inflammatory subtypes; and inducing macrophages to transition from a pro-inflammatory phenotype to an anti-inflammatory phenotype. In addition, the MSC-EVs in the treatment of Alzheimer’s disease also includes the clearance of Aβ, neuroprotective effects and as a potential drug delivery vehicle.

### The clearance of Aβ

Aβ is a hallmark pathological protein of AD, which are believed to be associated with neuronal damage and death. Once they exceed the clearance capacity of neuroglial cells, abnormal accumulation will lead to gradual decline in memory and cognitive dysfunction. It has been proven that clearing pathogenic proteins is beneficial for treating AD (90). MSCs-EVs can reduce the deposition of Aβ in the body through several different ways. Firstly, by inhibiting the expression of neutral sphingomyelinase-2 (nSMase2), the secretion function of pathological cells in AD patients can be reduced. This leads to a decrease in pathological exosomes and ultimately lowers the level of Aβ in the brain (91). Additionally, the reduction of nSMase2 can inhibit the conversion of sphingomyelin to ceramide, thereby increasing the level of sphingomyelin and promoting the secretion of exosomes from normal neurons. These exosomes can induce conformational changes in Aβ deposits, transforming them into fiber tissue without causing toxic effects on brain tissue. Surrounding microglial cells can uptake and degrade these fiber tissues, thereby reducing the amount of Aβ (92). Moreover, the surface of exosomes is rich in glycosphingolipids, which facilitate the binding of Aβ to exosomes. This characteristic enables exosomes to effectively serve as carriers for adsorbing Aβ and accelerating its removal from the body (93). Secondly, neprilysin (NEP) and insulin-degrading enzyme (IDE), as well as zinc metallopeptidase, are believed to be involved in the degradation of Aβ in the brain (94). As early as 2000, researchers injected radiolabeled synthetic Aβ peptides into the hippocampus of rats and observed that endogenous NEP could subsequently proteolytically degrade the peptides (95). In mice with NEP or IDE deficiencies, endogenous Aβ levels increased in a gene-dose-dependent manner (96, 97). Thirdly, research has found that in the human body, enkephalin is one of the enzymes in brain tissue that is most effective in breaking down and absorbing Aβ (98). Experiments have shown that when fat MSCs exosomes are added to the environment of AD model cells with high expression of Aβ-related proteins, the amount of Aβ detected in the cells and surrounding environment significantly decreases. This is due to the fact that fat MSCs exosomes are rich in enkephalinase levels that exceed the average (99). Hence, the crucial role of MSC-EVs in Aβ degradation highlights their potential in Alzheimer’s disease treatment.

### Neuroprotective effects of MSC-Evs

Another pathological hallmark of AD is synaptic dysfunction, which is directly associated with cognitive impairment. The experimental results from Mariana et al. (100) show that MSCs and their exosomes can protect hippocampal neurons and related synapses from damage caused by oxidative stress reactions resulting from Aβ deposition. Cui et al. (60) summarized the experimental results and speculated that MSC exosomes may improve learning and memory abilities in APP/PS1 double transgenic AD model mice by improving the function of damaged synapses and immune regulation at the site of injury. They found that exosomes extracted from mesenchymal stem cells subjected to hypoxic preconditioning significantly enhanced the expression of synaptic proteins (synapsin 1 and PSD95). The expression levels of synaptic proteins can to some extent reflect the function of synapses, and synapsin 1 and PSD95 are synaptic proteins involved in neural signal transmission and maintaining synaptic integrity. Another experiment showed that after fusion with neural cells, MSC exosomes can transfer miR-133b into neurons, promoting axonal repair and reducing neural damage caused by modeling. Additionally, MSC exosomes are rich in miR-17-92, and increasing their exogenous content can promote the generation of oligodendrocytes and axonal growth. In a transient cerebral ischemia mouse model, intravenous injection of exosomes with high expression of miR-17-92 enhanced neuronal plasticity and axonal growth speed compared to injection of normal MSC exosomes, achieving the effect of promoting neural function recovery (101).

### Potential drug delivery vehicle

The lipid bilayer structure of exosomes gives them excellent biocompatibility, supporting the loading of hydrophobic or hydrophilic drugs (102). Mesenchymal stem cell-derived exosomes can directly bind to membrane receptors through their exosomal exosome, allowing their contents to be internalized into target cells, or deliver bioactive substances to target cells through fusion with the plasma membrane. In addition, exosomes can easily cross the blood-brain barrier (BBB) and increase the concentration of drugs in the brain (103). Furthermore, exosome administration can avoid some complications, including intracranial infection, non-specific absorption, and drug toxicity, due to the low immunogenicity of exosomes (104). Previous studies have shown that exosomes can deliver drugs to the brains of AD mice (104). Furthermore, by using peptide-modified exosomes expressing the membrane protein Lamp2b, exosomes produced by engineered dendritic cells can bind to neuron-specific rabies virus glycoprotein (RVG) peptide, improving the cognitive function of AD transgenic mice (105).

## Blood exosomes as biomarkers of Alzheimer’s disease

In addition to potential therapeutic value, EVs can also serve as biomarkers, which is important in clinical applications. In particular blood exosomes, which are EVs secreted by living cells into the circulating blood, are regarded as a relatively noninvasive novel tool for monitoring brain physiology and disease states, and brain-derived exosomes in peripheral blood is an ideal biomarker for AD. A meta-analysis described the diagnostic performance of biomarkers of blood exosomes in AD (Registration No. CRD4200173498) (106). The findings revealed that individuals with preclinical Alzheimer’s disease, mild cognitive impairment, and Alzheimer’s disease exhibited elevated levels of core biomarkers, including Aβ1-42, P-T181-tau, P-S396-tau, and T-tau, in blood neuron-derived exosomes. Furthermore, there was an increase in molecules associated with other risk factors, such as C1q for neuroinflammation, P-S312-IRS-1 for metabolism disorder, HGF for neurotrophic deficiency, VEGF-D for vascular injury, and cathepsin D for autophagy-lysosomal system dysfunction. At the genetic level, the differential expression of REST, a transcription-related factor, and miR-132, a microRNA, also influenced RNA splicing, transport, and translation. These findings confirm the potential of the aforementioned core molecules and additional risk-related factors in blood exosomes as candidate biomarkers for preclinical and clinical Alzheimer’s disease. Consequently, these findings support the further development of exosome biomarkers for a clinical blood test for Alzheimer’s disease.

## Application of MSC-EVs in clinical practice and their advantages and limitations

There is presently an ongoing clinical trial, led by Ruijin Hospital affiliated with Shanghai Jiao Tong University, which aims to assess the safety and efficacy of utilizing allogeneic adipose-derived mesenchymal stem cells in patients with Alzheimer’s disease (www.clinicaltrials.gov). Although the clinical trial is still awaiting results, the therapeutic efficacy of MSC-EVs has shown promising outcomes in other conditions, such as pre-eclampsia (NCT03562715) and chronic ulcers (NCT04134676).

The advantages of MSC-EVs mainly lie in the following aspects: (1) The nanoscale MSC-EVs reduce vascular obstruction and are more capable of penetrating the blood-brain barrier (107); (2) MSC-EVs cannot replicate, avoiding uncontrolled division and reducing the risk of tumor formation during proliferation (108), as well as preventing mutations and DNA damage caused by cell transplantation (109); (3) MSC-EVs have low immunogenicity, making allogeneic applications possible (110); (4) Mesenchymal stem cells can produce a large amount of EVs, whose composition remains unchanged, facilitating storage and suitable for large-scale production (111). Apart from these advantages, the clinical application of MSC-EVs, especially in the context of AD, still faces certain limitations, primarily including: (1) the current methods for extracting MSC-EVs are time-consuming and inefficient, necessitating further exploration and research into efficient extraction methods that can be effectively applied in clinical settings; (2) Due to the different composition of cytokines in mesenchymal stem cell-derived extracellular vesicles from different sources, the clinical application relies on time-saving, cost-effective, and efficient methods. Further research is needed for the development of effective biomarkers for extracellular vesicles; (3) the specific mechanisms by which MSC-EVs regulate immune responses, promote Aβ degradation, and enhance axonal growth remain unclear and require further experimental investigation; (4) due to the complex biological composition of MSC-EVs, their safety when applied in animal models and the significance of specific therapeutic molecules within MSC-EVs warrant further attention.

## Conclusion

**Figure 1:**
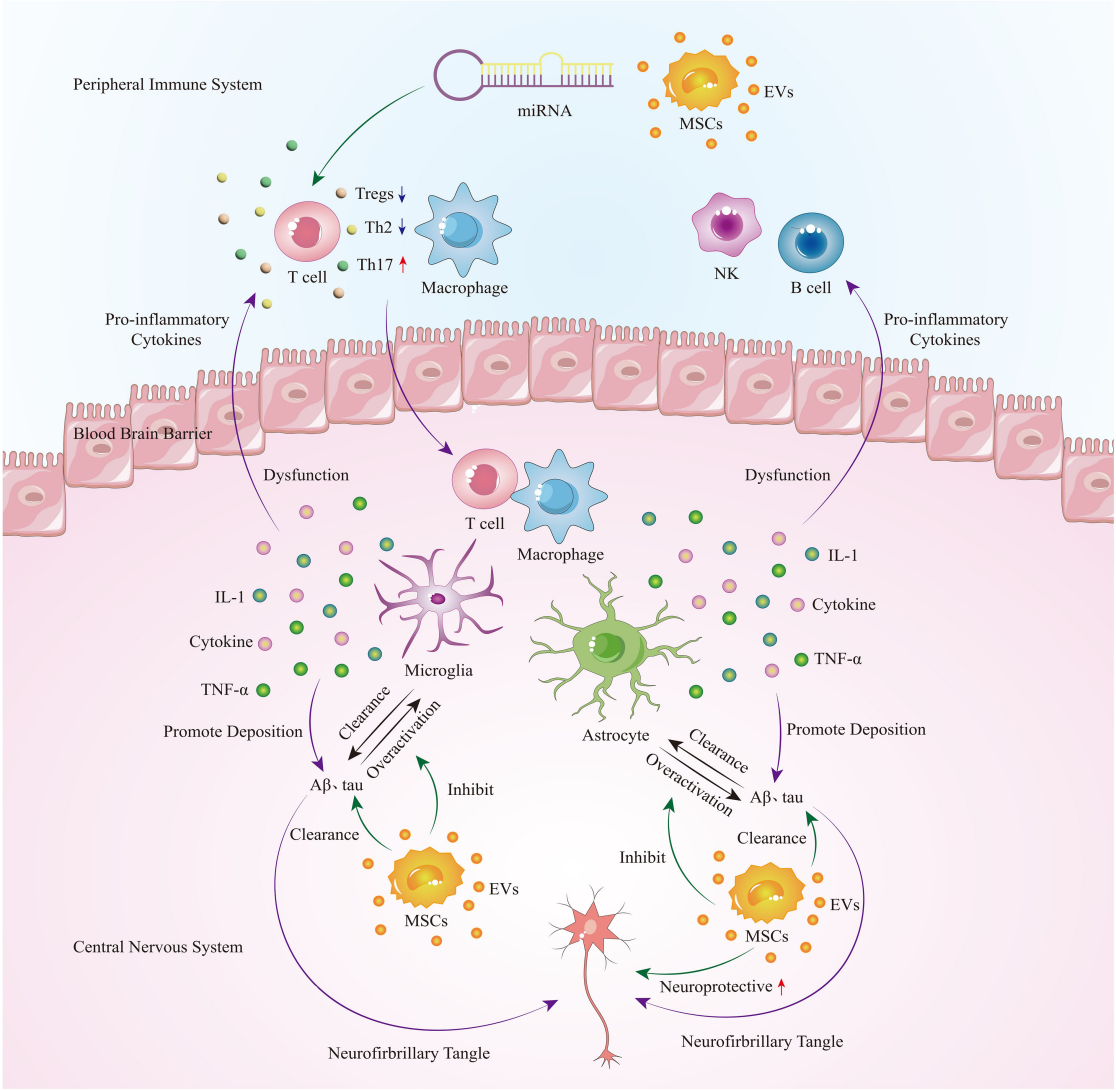
In the early stages of AD, immune cells, microglia, and astrocytes in the central nervous system are activated to clear protein deposits. However, as the disease progresses, glial cells become overactivated, leading to the secretion of a large number of pro-inflammatory cytokines. This not only exacerbates protein deposition but also damages the blood-brain barrier, allowing peripheral immune cells such as T cells and macrophages to infiltrate the brain, further exacerbating neuroinflammation and causing a vicious cycle. Extracellular vesicles derived from mesenchymal stem cells can regulate peripheral immune cells, inhibit overactive glial cells, and play a therapeutic role in Alzheimer’s disease by promoting neuroprotection and clearing protein deposits.

As a progressive neurodegenerative disease, Alzheimer’s disease currently lacks a cure. Previous research on the pathogenesis of Alzheimer’s disease has primarily focused on the abnormal accumulation of neurofibrillary tangles (NFTs) and amyloid plaques (Aβ). However, clinical trials targeting this mechanism have ended in failure, indicating that NFTs and Aβ are not the primary causes of Alzheimer’s disease. In recent years, studies have discovered that excessive immune response in the central nervous system may be a significant factor in protein deposition. In this pathological state, peripheral immune cells gather in the brain parenchyma through a compromised blood-brain barrier, further exacerbating the progression of Alzheimer’s disease. Mesenchymal stem cell-derived extracellular vesicles (MSC-EVs), as a cell-free therapy, have demonstrated excellent immunomodulatory effects on both central nervous system immune cells and peripheral immune cells. They have also shown two major benefits in Alzheimer’s disease: clearing protein deposits and neuroprotection (**Figure 1**). Compared to the MSC - Evs as drug delivery carrier alone, directly isolated MSC-EVs retain natural substances and surface markers, which can minimize immune rejection and other potential complications. Engineering vesicles can by modifying the composition of vesicle or surface characteristics, load specific drugs or therapeutic molecules, so as to realize precise targeting and controlled release, but the engineering process can be complex and may alter the natural properties of the vesicles. The choice between direct isolation of MSC-EVs or preparation of engineered vesicles as therapeutic interventions depends on the specific application and desired outcomes. Further research and clinical trials are needed to determine which approach is more effective and practical in different therapeutic contexts. Currently, there is an ongoing study investigating the safety and efficacy of MSC-EVs in treating Alzheimer’s disease. It is believed that in the near future, further exploration of its therapeutic mechanisms and optimization of treatment strategies will provide more effective treatment options for Alzheimer’s disease patients.

## Author contributions

YY: Funding acquisition, Writing – original draft, Writing – review & editing. MG: Writing – original draft. WS: Writing – review & editing. YG: Writing – review & editing. YL: Writing – review & editing. WY: Writing – review & editing. XZ: Supervision, Writing – review & editing. XL: Conceptualization, Supervision, Writing – review & editing.
